# Efficacy of valgus osteotomy in treating nonunion of femoral neck fractures post-internal fixation

**DOI:** 10.3389/fsurg.2025.1526384

**Published:** 2025-02-17

**Authors:** Haohao Xu, Shenda Yang, Yuanyuan Zheng, Ao Tian, Ruofei Wu, Xiaoyu Zhang, Yujiang Mao

**Affiliations:** ^1^ Department of Orthopedic Trauma, Beijing Jishuitan Hospital, Capital Medical University, The 4th Medical College of Peking University, Beijing, China; ^2^ Department of Anesthesiology, Beijing Jishuitan Hospital, Capital Medical University, The 4th Medical College of Peking University, Beijing, China; ^3^ Department of Orthopedics, Beidahuang Industry Group General Hospital, Harbin, China; ^4^ Department of Orthopedics, The Beian Hospital of Beidahuang Group, Beian, China

**Keywords:** femoral neck fractures, valgus intertrochanteric osteotomy, ununited, fractures, internal fixation

## Abstract

**Background:**

Nonunion of femoral neck fractures following internal fixation presents a significant challenge, particularly in young patients. This study evaluated the efficacy of valgus osteotomy in treating femoral neck fracture nonunion after failed internal fixation in young adults.

**Methods:**

A retrospective analysis was conducted including a total of 23 male patients with a mean age of 32.9 years, who underwent valgus osteotomy for femoral neck fracture nonunion. The procedure involved lateral closing wedge osteotomy at the subtrochanteric level, followed by fixation with a dynamic hip screw (DHS). The postoperative follow-up period exceeded one year.

**Results:**

Significant improvements in biomechanical parameters were observed. The mean neck-shaft angle increased from 118.91° to 139.26° (*p* < 0.001), whereas the Pauwels angle decreased from 62.07° to 46.91° (*p* < 0.001). The median Harris Hip Score improved from 63 to 96 (*p* < 0.001). Limb length demonstrated a trend toward improvement, increasing from a median of 73.30–74.50 cm, although this was not statistically significant (*p* = 0.077). Union was achieved in 22 of 23 patients, with a median healing time of 8.5 months.

**Conclusion:**

Valgus osteotomy is an effective treatment for femoral neck fracture nonunion in young adults, offering improved biomechanics, good union rates, and enhanced functional outcomes. This technique is a viable option for preserving the native hip joint in challenging patients.

## 1 Introduction

Femoral neck fractures are a common type of hip injury, typically caused by traffic accidents or falls (1). The blood supply to the femoral head may be compromised, leading to nonunion of the fracture or avascular necrosis of the femoral head (2). Internal fixation is the standard treatment for femoral neck fractures in young patients; however, owing to insufficient blood supply, poor mechanical stability, and excessive shear stress at the fracture line, approximately 10%–30% of patients experience nonunion post-surgery (3).

For older patients with nonunion, hip replacement surgery can rapidly alleviate pain, partially restore function, reduce the risk of long-term bed confinement, and effectively address nonunion issue (4). However, younger and middle-aged patients often require higher long-term hip function, making head-preserving treatment strategies such as revision internal fixation, proximal femoral osteotomy, and valgus osteotomy crucial for maintaining mobility and quality of life. Valgus osteotomy can optimize the biomechanical environment of the hip joint and reduce the Pauwels’ angle at the fracture end of the femoral neck, thereby promoting fracture healing. This method can precisely reconstruct biomechanical characteristics conducive to healing, preserve the femoral head, promote healing and functional recovery, and delay or avoid total hip replacement (5).

Although some studies have reported the application of valgus osteotomy in femoral neck fractures (5), its application in nonunion after internal fixation lacks widespread clinical data. Therefore, this study aimed to present our experience using valgus osteotomy to treat nonunion in patients with femoral neck fractures after internal fixation and to evaluate its clinical effectiveness.

## 2 Materials and methods

This study was approved by the Ethics Committee of the Beijing Jishuitan Hospital (No. 2023129-00). Data on patients who underwent valgus osteotomy for nonunion of femoral neck fractures following hollow nail surgery were collected from the Department of Traumatology and Orthopedics at Beijing Jishuitan Hospital between July 2017 and November 2022. The inclusion and exclusion criteria were as follows.

Inclusion criteria: (1) initial surgery involving internal fixation for femoral neck fracture, (2) clinical and radiological evidence consistent with a diagnosis of postoperative nonunion of the femoral neck fracture, (3) age between 14 and 55 years, and (4) closure of the femoral head epiphysis in minor patients (skeletal maturity). Exclusion criteria: (1) MRI showing definite avascular necrosis of the femoral head with collapse, and (2) clinical and laboratory evidence of acute or chronic infection.

### 2.1 Preoperative preparation

**Figure 1:**
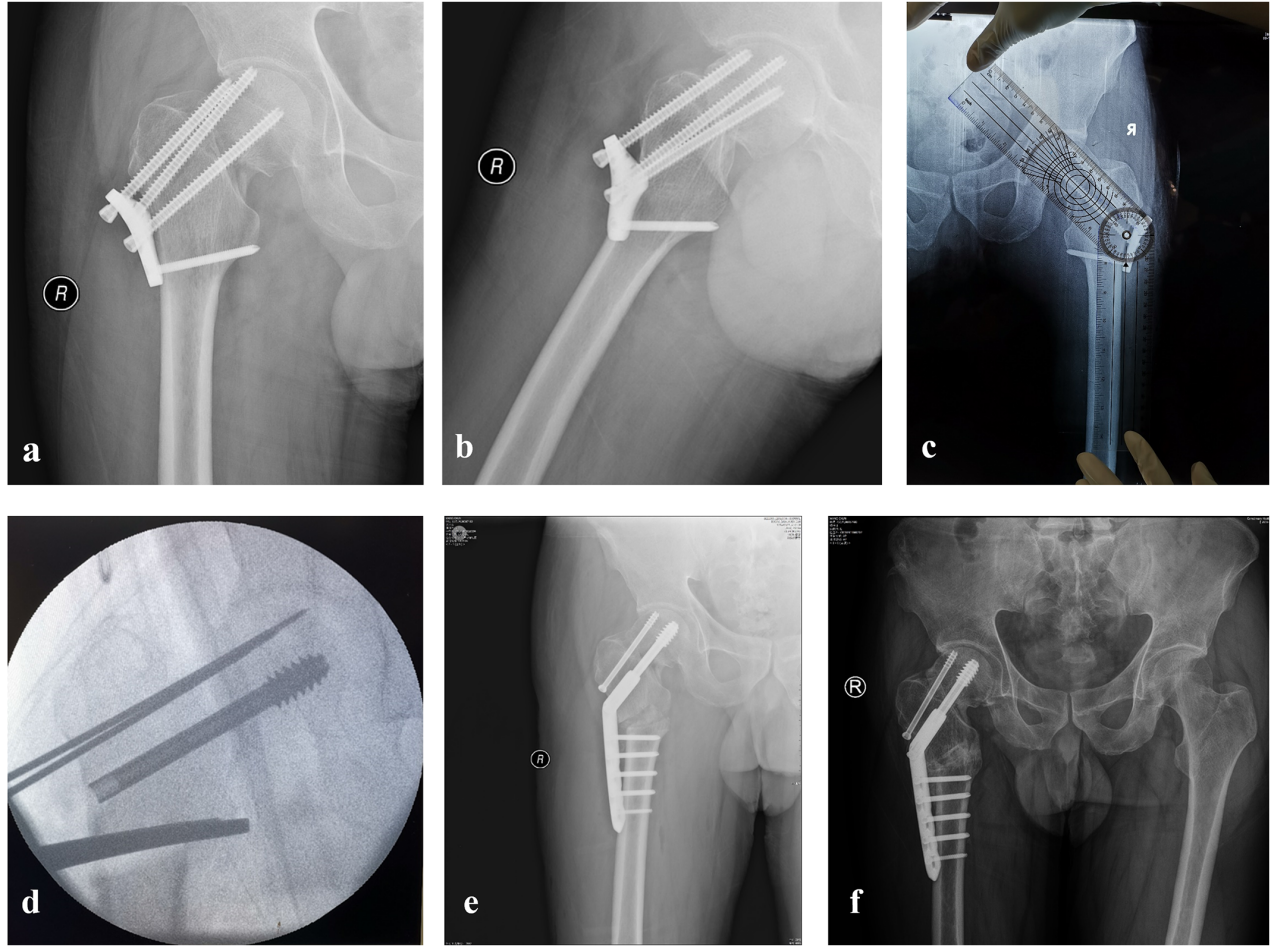
Valgus osteotomy surgery situation; **(a,b)**, the preoperative situation of valgus osteotomy surgery; **(c,d)**, intraoperative planning; **(e)** postoperative; **(f)**, 8 months postoperative.

**Figure 2:**
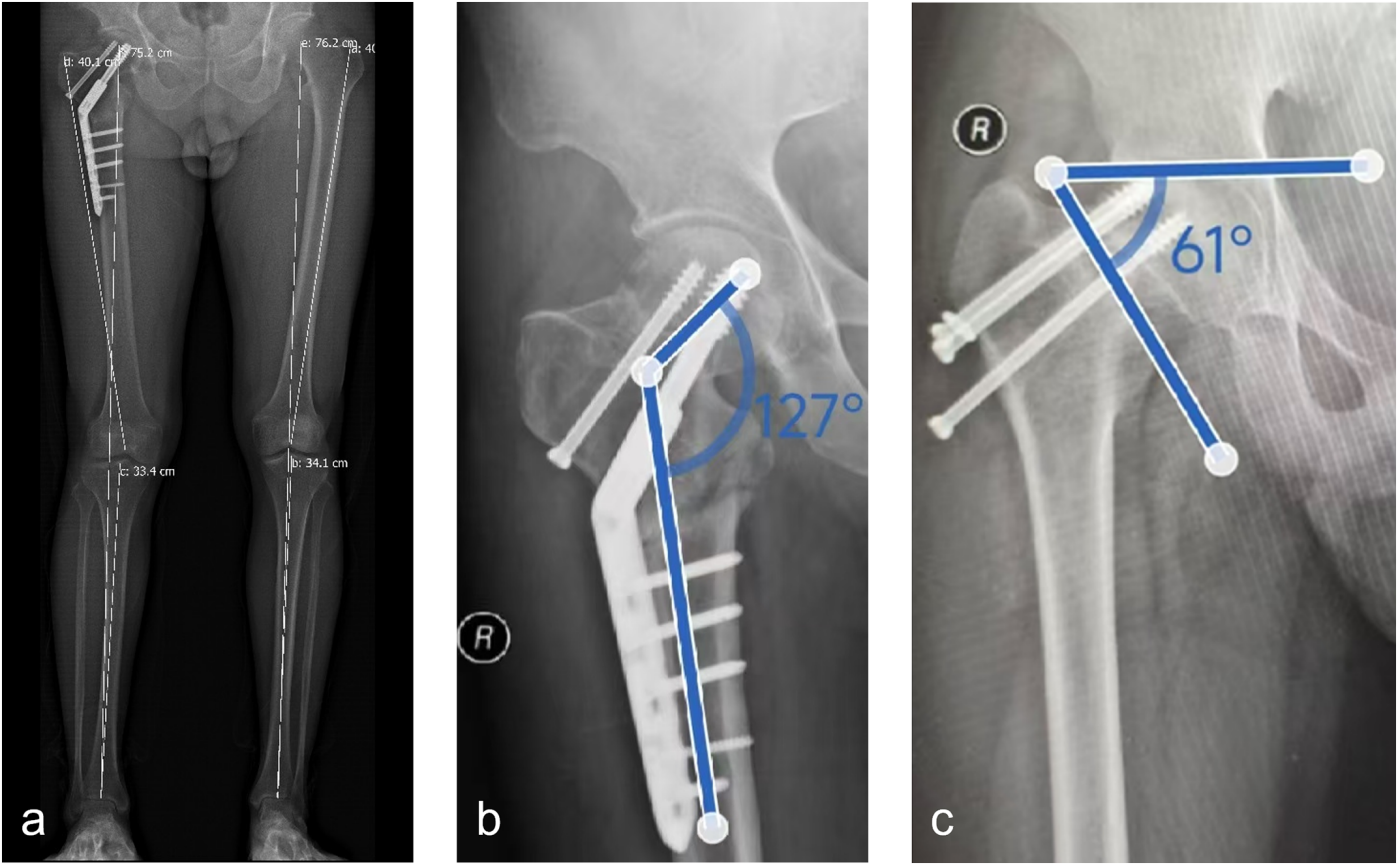
Test methods for the mean neck-shaft angle, pauwels angle, and limb length. **(a)** Limb length: Calculated as the sum of the femoral length (from the femoral head center to the distal condyle) and the tibial length (from the tibial plateau to the medial malleolus); **(b)** Neck-shaft angle: Measured on an anteroposterior radiograph of the hip as the angle formed by the intersection of the femoral shaft axis and the femoral neck axis; **(c)** Pauwels angle: Measured on an anteroposterior radiograph of the hip as the angle formed between the distal fracture line of the femoral neck and the line connecting the bilateral iliac crests.

Preoperative anteroposterior hip radiographs were used to measure the neck-shaft angle, Pauwels angle, and length discrepancy between the affected and contralateral limbs (Figures 1a,b). A comprehensive assessment was conducted based on the presence of coxa vara, preoperative neck-shaft angle, fracture line, Pauwels angle, femoral neck shortening, and limb length discrepancy to determine individualized correction angles and osteotomy sites. Limb length: measured clinically or radiographically. In clinical assessment, it is defined as the distance between the anterior superior iliac spine and the medial malleolus. In radiography, it is calculated as the sum of the femoral length (from the femoral head center to the distal condyle) and the tibial length (from the tibial plateau to the medial malleolus) (Figure 2a). The neck-shaft angle, defined as the angle formed by the intersection of the femoral shaft axis and the femoral neck axis, was measured on an anteroposterior radiograph of the hip (Figure 2b). Pauwels angle: measured on an anteroposterior radiograph of the hip; this angle is formed between the distal fracture line of the femoral neck and the line connecting the bilateral iliac crests (Figure 2c). The general principle was to reduce the postoperative Pauwels angle to below 40° and increase the postoperative neck-shaft angle to approximately 140°, with a correction angle of 15°–30°. The entry point and trajectory of the screw on the lateral wall of the proximal femur were planned based on the 135° angle of the DHS screw (Figure 1c). The osteotomy site and angle were determined intraoperatively as follows: After confirming the insertion of the DHS lag screw, the osteotomy was initiated 1.5 cm below the lateral cortex of the screw, with the osteotomy plane oriented obliquely toward the middle to lower third of the lesser trochanter.

### 2.2 Surgical procedure

Under spinal anesthesia, the patient was positioned supine on a traction table with the affected limb slightly adducted and internally rotated. Traction was applied along the long axis of the body. A 15 cm longitudinal incision was made on the proximal-lateral side of the affected thigh along the posterior edge of the vastus lateralis muscle to expose the proximal femur. The tail caps of the hollow nails were exposed and removed. Using intraoperative images and typical landmarks, such as the greater trochanter, lesser trochanter, lateral cortex, femoral shaft axis, and femoral neck axis, three guide pins were inserted based on the preoperative correction angles: one for the hollow nail, one for the DHS screw, and one for guiding the osteotomy plane and angle (Figure 1d). After confirming the screw paths using biplanar radiography, three guide pins were inserted into the target positions. The lengths were measured, and the hollow nail and DHS screw were sequentially screwed to the appropriate depths. Intertrochanteric osteotomy, guided by a third pin, was subsequently performed. The osteotomy was initiated 1.5 cm below the lateral cortex of the DHS lag screw and extended obliquely toward the middle-lower third of the lesser trochanter. A DHS plate was then placed, and distal plate alignment with the lateral cortex of the femur was achieved using reduction forceps for valgus correction. Cortical and locking screws were used to secure the DHS plates. A stabilizing bolt was inserted at the end of the main nail to apply mild compression to the nonunion site, with no bone grafting required at the femoral neck nonunion site or medial opening of the intertrochanteric osteotomy.

### 2.3 Postoperative management

On the day of surgery, after recovery from anesthesia, the patients were instructed to perform ankle pump exercises and isometric quadriceps contractions. On the first postoperative day, the patients were allowed to sit at the bedside and begin heel-touch hip flexion and extension exercises. One week postoperatively, the patients began walking with crutches and touching their toes on the ground. Radiographs were reviewed monthly until fracture healing was confirmed. Once healed, the patient gradually progressed to full weight-bearing on the affected limb (Figures 1e,f). Follow-up anteroposterior and lateral hip radiographs were performed 1-year post-surgery.

### 2.4 Observation and outcome evaluation indicators

The key observation indicators included operation time, intraoperative blood loss, perioperative complications, and changes in the Pauwels angle, neck-shaft angle, and limb length discrepancy preoperatively and postoperatively. Additionally, the postoperative fracture healing time and complications were recorded. During follow-up, the Harris Hip Score system was used to evaluate the hip joint function of the affected limb. The system provides a total score of 100 points, with the following categories: >90 points excellent, 80–90 points good, 70–79 points fair; and <70 points, poor. Radiological data were measured by two attending orthopedic trauma surgeons, and the average value was recorded.

### 2.5 Statistics

For data that followed a normal distribution, the results were expressed as the mean ± standard deviation (mean ± SD), and preoperative and postoperative changes were compared using paired t-tests. For data that did not follow a normal distribution, the results were expressed as median and interquartile range [median (IQR)] and compared using the Wilcoxon signed-rank test. Categorical data were presented as frequencies and percentages, and differences between groups were compared using the chi-square test or Fisher's exact test. Additionally, Kaplan–Meier (KM) curves were used to describe the postoperative healing of femoral neck fractures. Data processing and analysis were performed using R and SPSS software, with statistical significance set at *p* < 0.05.

## 3 Results

A total of 23 male patients with a mean age of 32.9 years (standard deviation, 12.5) participated in the study. The mean body mass index (BMI) was 23.83 (standard deviation, 4.43). The Garden classification of fracture types included 3 cases of Type I, 6 cases of Types I and II, 5 cases of Type III, and 9 cases of Type IV. Fourteen patients sustained femoral neck fractures due to high-energy injuries, such as traffic accidents or falls from a height, while nine patients experienced fractures from low-energy injuries. Among the internal fixation failure cases, there were eight cases of implant failure, 14 cases of screw loosening, and one case of nonunion. Additionally, one patient had a history of diabetes, one had hypertension, five had a history of smoking, one had a history of alcohol use, and one had a history of both smoking and alcohol use (Table 1).

**Table 1:** Data relating to patient demographics and first surgery.

Characteristic	*N* = 23
Sex, *n* (%)	
Male	23 (100%)
Age (year; M ± SD)	32.9 ± 12.5
BMI(M ± SD)	23.83 ± 4.43
Internal fixation failure	
Screw backout	8
Nonunion	14
Failure	1
Fracture type	
I	3
II	6
III	5
IV	9
Type of injury	
High-energy injury	14
Low-energy injury	9
Chronic disease history	
Diabetes	1
Hypertension	1
Adverse lifestyle history	
Smoking	5
Alcohol consumption	1
Smoking and alcohol consumption	1

**Figure 3:**
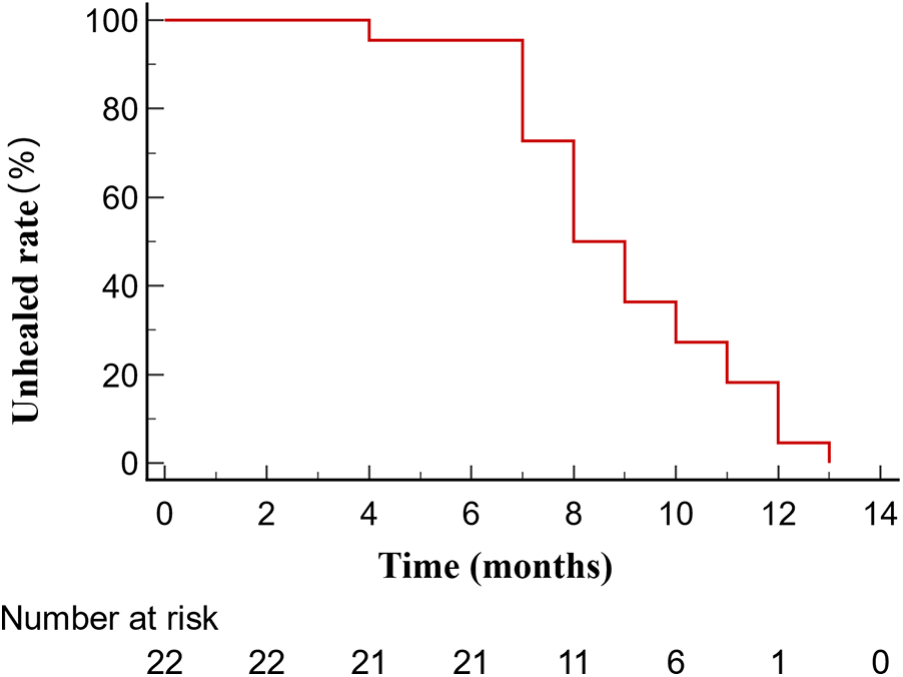
Kaplan–Meier curves describe the healing situation of femoral neck fractures after valgus osteotomy surgery.

The average blood loss during valgus osteotomy was 395.65 ml(standard deviation of 208.30 ml), with a median operation time of 165 min (interquartile range of 127.50–180.00 min). The median interval between surgeries was 8 months (interquartile range, 5–12 months), and the median time for patients to walk without crutches was 5 months (interquartile range, 3–6 months). Except for one patient, all patients achieved fracture union with a median healing time of 8.5 months (interquartile range, 7–11 months; Table 2, Figure 3).

**Table 2:** Valgus intertrochanteric osteotomy and prognosis.

Characteristics	*N* = 23
Blood loss (ml, M ± SD)	395.65 ± 208.30
Surgery duration [min; median (IQR)]	165.00 [127.50, 180.00]
Time interval between surgeries [month; median (IQR)]	8 [5, 12]
Time to walk without crutches [month; median (IQR)]	5 [3, 6]
Healing time of femoral neck fracture [month; median (IQR)]	8.5 [7, 11]

The postoperative neck-shaft angle significantly increased from a preoperative average of 118.91° (standard deviation, 7.27°) to a postoperative average of 139.26°(standard deviation, 5.57°) (*p* < 0.001). The Pauwels angle decreased from a preoperative average of 62.07° (standard deviation, 7.33°) to a postoperative average of 46.91° (standard deviation, 9.36°), showing significant improvement (*p* < 0.001). The Harris Hip Score significantly improved from a preoperative median of 63 points (interquartile range, 60–68 points) to a postoperative median of 96 points (interquartile range, 86–99 points; *p* < 0.001) (Supplementary Table S1).

**Figure 4:**
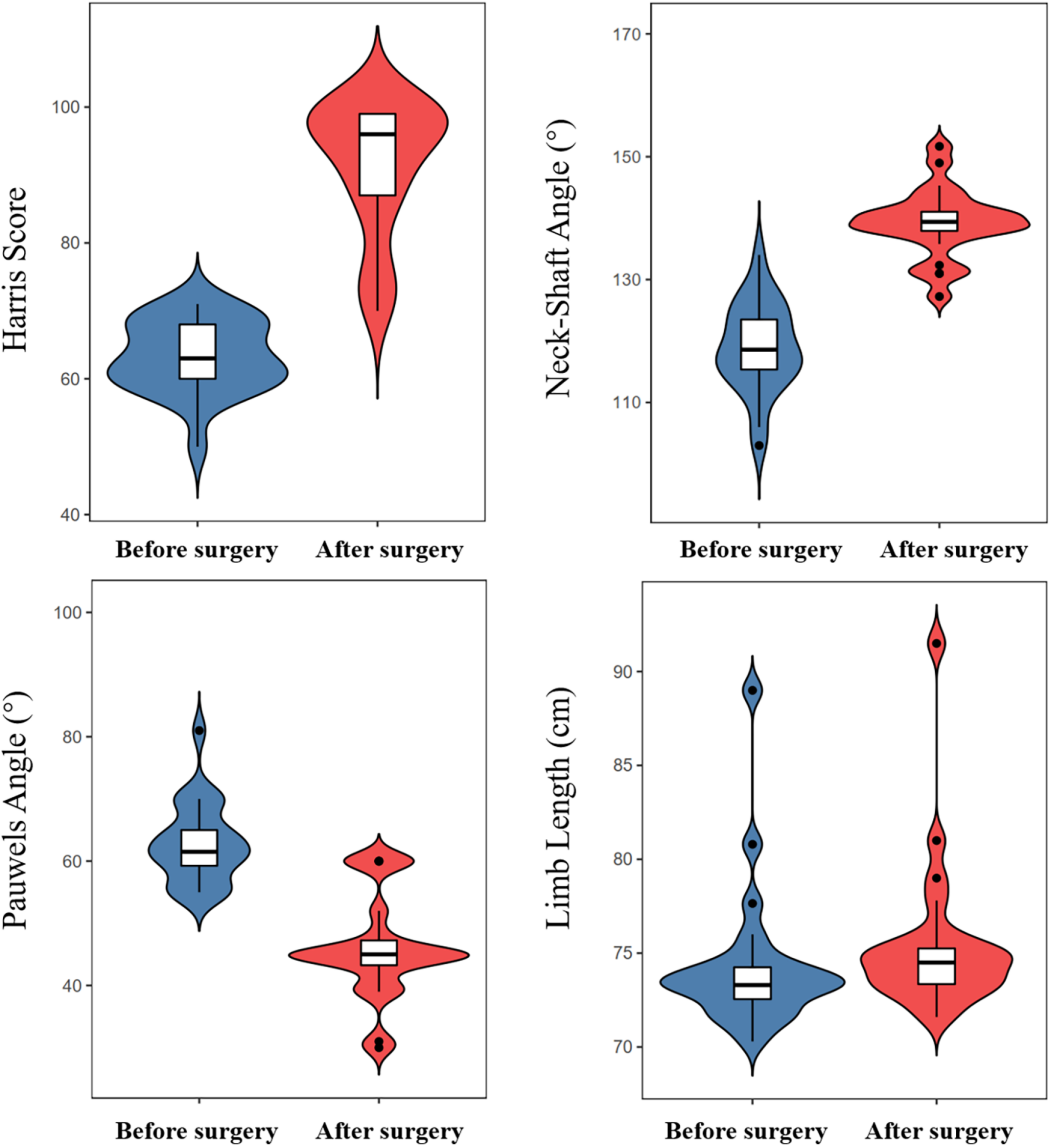
Violin plot showing the changes in key indicators after surgery.

Postoperative limb length increased from a preoperative median of 73.30 cm (interquartile range of 72.55–74.25 cm) to a postoperative median of 74.50 cm (interquartile range of 73.35–75.25 cm); however, the *p*-value was 0.077, which was not statistically significant (Table 3, Figure 4).

**Table 3:** Effectiveness of abduction osteotomy.

Variable	Before surgery	After surgery	*p*
Neck-shaft angle (degrees, M ± SD)	118.91 ± 7.27	139.26 ± 5.57	<0.001
Pauwels angle (degrees, M ± SD)	62.07 ± 7.33	46.91 ± 9.36	<0.001
Harris score median [IQR]	63 [60, 68]	96 [86, 99]	<0.001
Limb length [cm; median (IQR)]	73.30 [72.55, 74.25]	74.50 [73.35, 75.25]	0.077

## 4 Discussion

Nonunion of femoral neck fractures is a significant complication following internal fixation, occurring in 10%–33% of young to middle-aged patients (3). Blood supply to the femoral neck primarily originates from the circumflex arteries beneath the femoral head, which are vulnerable to damage during fractures. Significant displacement can further exacerbate vascular injury, leading to insufficient blood supply, impaired healing, and an increased risk of nonunion (2).

The biomechanical characteristics of femoral neck fractures also play a crucial role. In young and middle-aged patients, these fractures frequently result from high-energy injuries, leading to a relatively vertical fracture line (Pauwels Type III) (6). This fracture pattern experienced high shear forces that were detrimental to healing. Additionally, femoral neck fractures are often accompanied by varus deformities, which further increase the shear stress at the fracture site (6).

Moreover, internal fixation can affect fracture healing. Improper reduction, unstable fixation, and inappropriate fixation device selection may contribute to nonunion (7, 8). Although hollow screw fixation is less invasive, its limited resistance to rotational forces allows for micromovements at the fracture ends, hindering healing (9). Despite the generally good bone quality in younger patients, their higher activity levels necessitate more robust fixation. Factors such as smoking, alcohol consumption, and poor nutrition can adversely affect healing.

In this study, all femoral neck fracture patients with nonunion treated at our center during the study period were male. This phenomenon may be attributed to the higher likelihood of males engaging in high-risk occupations and activities, such as manual labor and driving, which increases their exposure to high-energy trauma, such as traffic accidents. Consequently, the incidence of femoral neck fracture is higher in males. Furthermore, after internal fixation surgery, male patients who are often engaged in physically demanding jobs may resume physical work earlier, leading to increased mechanical stress at the fracture site and a higher risk of nonunion.

### 4.1 Treatment options for nonunion of femoral neck fractures

Current treatment options for femoral neck fracture nonunion include various strategies tailored to the patient's age, fracture characteristics, and expected outcomes. Total hip arthroplasty (THA) is a well-accepted procedure that provides rapid pain relief and restores hip function in older patients, allowing for early mobilization (10). However, in younger patients with longer life expectancies, the long-term outcomes of artificial joints remain uncertain and may require multiple revision surgeries (11). Therefore, joint-preserving treatments are often preferred for young and middle-aged patients.

In some cases of early nonunion, simple revision of the internal fixation may be a viable option (12). This minimally invasive approach is relatively straightforward but may not address underlying biomechanical issues, such as varus deformities or implant-related instability, leading to high recurrence risks.

Vascularized fibular or iliac bone grafting can enhance the local blood supply and provide structural support, creating a favorable biological environment for healing. However, this technique is technically demanding, requires prolonged surgery, and increases the risk of complications (13). Furthermore, non-vascularized bone grafting delays the need for THA in early stage osteonecrosis; however, 21% of patients still progress to THA within a 50-month follow-up, highlighting the limitations of this joint-preserving strategy (12, 13).

Proximal femoral valgus osteotomy (PFVO) is a surgical method designed to improve the biomechanical environment of the fracture site. By altering the orientation of the fracture line, shear forces are converted into compressive forces that promote fracture healing. This procedure corrects varus hip deformities and restores normal anatomical relationships (5). Although the risk of infection is higher in patients undergoing multiple surgeries, valgus osteotomy is generally less invasive and technically simpler, with a lower risk of infection due to its extra-articular approach (14). Jain and Angelini emphasized the effectiveness of valgus osteotomy in achieving union rates exceeding 85% while preserving the femoral head (12, 13). However, mild gait abnormalities and abductor weakness may occur because of changes in biomechanical alignment (12).

### 4.2 Advantages of valgus osteotomy

The primary advantage of valgus osteotomy is its ability to significantly improve the biomechanical environment of the fracture site. By changing the fracture line from vertical to horizontal, the procedure converts the shear forces into compressive stress, which is conducive to healing (15). This principle, first introduced by Pauwels, has been validated in multiple studies, emphasizing the importance of biomechanical optimization for fracture healing (14, 15). Our study demonstrated that the postoperative Pauwels angle decreased from an average of 62.07 ± 7.33°–46.91 ± 9.36°, creating a more favorable biomechanical environment for healing. Reducing the Pauwels angle helps optimize the biomechanical environment at the fracture site by decreasing the proportion of the shear force and increasing the compressive force, which is more conducive to fracture healing. Importantly, the patient's body weight plays a critical role in determining the absolute magnitudes of these forces. Heavier patients experience greater overall shear and compressive forces on the femoral neck, which may increase the risk of delayed union or nonunion. Our findings highlight the importance of preoperative imaging in evaluating Pauwels angles and fracture types, as well as patient-specific factors such as body weight, to guide surgical planning and improve outcomes.

In addition to optimizing fracture biomechanics, valgus osteotomy restores the normal anatomical relationships of the hip, particularly the neck-shaft angle. Misalignment of the femoral neck, often observed in varus deformities, was corrected to achieve a more physiological neck-shaft angle (14). Said reported that their patients achieved a postoperative neck-shaft angle of 139° (range: 125°–160°), which closely approximates the normal anatomical values (15). Similarly, in our study, the neck-shaft angle increased from a preoperative average of 118.91° ± 7.27° to a postoperative average of 139.26° ± 5.57°, approaching normal anatomical angles. This restoration improves hip biomechanics, reduces long-term complications, and preserves future treatment options.

Valgus osteotomy promotes fracture healing and significantly enhances patient function. Our study demonstrated an improvement in the Harris Hip Score, increasing from a preoperative median of 63 (60, 68) to a postoperative median of 96 (86, 99). This improvement aligns with findings from Schwartsmann, who reported an average Harris Hip Score of 81.2 points (±7.2) in their long-term follow-up of valgus osteotomy patients (14), and from Said, where 61% of patients were pain-free, and 72% could walk without limping at the final follow-up (15). Some patients exhibited gait abnormalities post-osteotomy, attributed to changes in limb mechanical alignment and the abductor lever arm owing to the increased neck-shaft angle. Gait abnormalities were more pronounced and frequent in older patients but rarely observed in younger patients, which is consistent with previously reported results (15). This significant functional improvement underscores the advantages of valgus osteotomy in restoring hip function.

Compared with other treatments, such as THA, which may have limitations in functional recovery, valgus osteotomy poses a lower risk of complications. Compared to vascularized bone grafts, valgus osteotomy involves a shorter surgical time and less trauma, thereby reducing the risk of surgery-related complications (16).

### 4.3 Characteristics of the valgus osteotomy technique

Osteotomy was performed in the lower third of the lesser trochanter, which has several advantages. First, it avoids disturbing the greater trochanteric region, thereby preserving abductor muscle function. Second, the bone quality at this site is favorable for bone healing. Finally, the risk of lateral wall fractures was minimized by maintaining a safe distance from the DHS. An open oblique osteotomy technique was used instead of the traditional lateral closed-wedge removal osteotomy. This technique was chosen because the target population in this study consisted of patients with femoral neck fracture nonunion rather than coxa vara deformity. Open oblique osteotomy is simpler to perform and allows flexible intraoperative adjustment of the valgus correction angle. Additionally, it avoids limb shortening caused by lateral wedge bone removal, which is particularly important in patients with limb length discrepancies resulting from nonunion. This approach allows for greater correction as the oblique osteotomy line facilitates slight sliding to maintain good bone contact with the medial cortex remaining largely intact, thereby aiding in stability and avoiding the need for bone removal, which facilitates limb length restoration.

### 4.4 Potential failure factors and preventive measures of valgus osteotomy

Despite the positive outcomes of valgus osteotomy for the treatment of nonunion of femoral neck fractures in young and middle-aged patients, several factors can lead to treatment failure. An insufficient correction angle is a major cause of surgical failure. An inadequate valgus angle may fail to sufficiently improve the biomechanical environment at the fracture site, resulting in persistent nonunion (17). To prevent this, detailed preoperative planning is essential. The choice and placement of internal fixation devices are crucial for surgical success. Unstable fixation can lead to continuous micromovements at the fracture ends, thereby hindering fracture healing. Using DHS as the primary fixation, along with an anti-rotational screw when necessary, provides adequate stability while preserving the sliding compression effects.

Although valgus osteotomy can enhance the mechanical environment at the fracture site, severe impairment of the local blood supply can significantly affect healing. Detailed imaging evaluations should be conducted preoperatively to exclude severe avascular necrosis of the femoral head and to assess and preserve blood supply. It is crucial that the osteotomy technique protects the surrounding soft tissues as much as possible to maintain local blood supply.

Patient compliance, bone quality, and overall health significantly affect surgical outcomes. Comprehensive preoperative evaluation and appropriate health guidance are essential. During postoperative rehabilitation, patients should be encouraged to eliminate harmful lifestyle habits, such as smoking, and should be closely monitored for adherence.

This study had several limitations. First, it was retrospective and involved a relatively small sample size, elucidating the need for future prospective multicenter studies with larger cohorts to validate our findings. Additionally, all patients in this study were male, which limits the generalizability of our findings. Future studies should aim to include a more diverse patient population, not only to confirm the outcomes of valgus osteotomy, but also to explore how factors such as occupational and behavioral differences, such as the tendency for males to return to physical labor earlier, might influence fracture healing and treatment success. These insights could help inform more tailored postoperative care strategies to optimize recovery in different patient groups. Although all included minors had closed growth plates, it remains unclear whether their recovery differs from that of young and middle-aged adults, and this warrants further investigation.

The mean follow-up period in our study was 18.6 months, which was adequate for assessing fracture healing and early functional recovery. However, it does not capture long-term outcomes, including complications such as avascular necrosis or the progression of degenerative changes. Further follow-up studies are required to better understand the durability of valgus osteotomies and their potential complications. Moreover, changes in femoral neck morphology resulting from osteotomy may pose challenges for subsequent procedures, such as THA. Identifying patients who are most likely to benefit from this procedure while minimizing long-term risks remains an important area for future research.

## 5 Conclusion

Valgus osteotomy is an effective approach for treating nonunion following internal fixation in young and middle-aged patients. This procedure optimizes the biomechanical environment at the nonunion site, and when combined with stable internal fixation, consistently yields favorable healing outcomes. It is a relatively straightforward technique that minimizes the surgical time, reduces blood loss, and aids in restoring limb length. Future research should investigate individualized osteotomy strategies, including precise osteotomy angle design based on finite element analysis, and the potential benefits of combining valgus osteotomy with biological enhancement techniques, such as PRP and BMP, to further improve surgical outcomes.

## Data Availability

The raw data supporting the conclusions of this article will be made available by the authors, without undue reservation.
