# Supplementary material for: Efficacy of valgus osteotomy in treating nonunion of femoral neck fractures post-internal fixation
Source: Front Surg. 2025 Feb 17;12:1526384. doi: 10.3389/fsurg.2025.1526384 (PMC11872901; doi:10.3389/fsurg.2025.1526384)
Supplement: Supplementary file 1 [file Table1.docx]

| Table S1 Mean neck-shaft angle and Pauwels angle for the 23 patients | | | | |
| --- | --- | --- | --- | --- |
| Patient No. | Preoperative Neck-Shaft Angle (°) | Postoperative Neck-Shaft Angle (°) | Preoperative Pauwels Angle (°) | Postoperative Pauwels Angle (°) |
| 1 | 117.5 | 138 | 70 | 44 |
| 2 | 118.6 | 138 | 64 | 45 |
| 3 | 125 | 139.4 | 56 | 30 |
| 4 | 123 | 145.3 | 60 | 31 |
| 5 | 115.7 | 135.8 | 81 | 48 |
| 6 | 116 | 141 | 61 | 60 |
| 7 | 119 | 131 | 70 | 52 |
| 8 | 123 | 143 | 70 | 39 |
| 9 | 128 | 151.7 | 55 | 45 |
| 10 | 116.4 | 138.5 | 64 | 45 |
| 11 | 119.3 | 139.5 | 61 | 40 |
| 12 | 134 | 138 | 55 | 43 |
| 13 | 127 | 149 | 55 | 39 |
| 14 | 122.4 | 141 | 65 | 45 |
| 15 | 103 | 141 | 65 | 45 |
| 16 | 124 | 139 | 45 | 68 |
| 17 | 106 | 131 | 62 | 60 |
| 18 | 115 | 141.09 | 60.5 | 45 |
| 19 | 112.53 | 132.35 | 63 | 45 |
| 20 | 128 | 140.41 | 59 | 45 |
| 21 | 116.62 | 143.83 | 61 | 60 |
| 22 | 113.53 | 137.84 | 55 | 45 |
| 23 | 111.35 | 127.25 | 70 | 60 |
